# Radical Improvements to the Fourth Year Medical Student Experience Whilst on Placement at Black Country Healthcare NHS Foundation Trust

**DOI:** 10.1192/bjo.2025.10373

**Published:** 2025-06-20

**Authors:** Joanna Pegg, Amar Hujan, Thomas Lowde, Annabel Ariyathurai, Devika Patel

**Affiliations:** 1 Black Country Healthcare NHS Foundation Trust, Dudley, United Kingdom; 2 Birmingham and Solihull Mental Health NHS Foundation Trust, Birmingham, United Kingdom

## Abstract

**Aims:** Feedback was collected from students at Black Country Healthcare Foundation Trust to survey their satisfaction with their psychiatry placement which has been changed from nine weeks to five and had moved away from a carousel system to allow independent learning for students. Primary aim was to assess student satisfaction with secondary aims to improve access to a broader knowledge of sub-specialities.

**Methods:** Resident doctors collaborated with the medical student tutor and an initial feedback from students was gained. A plan was formulated to deliver a trust-specific 3-week teaching programme. We created a new improved handbook with opportunities for students to attend sub-specialities.

Suggestions for improvement were collated from each set of new medical students and some from previous years with this updated at the end of each rotation. We also had regular meetings with stakeholders to monitor progress and appointed medical student champions as a way of linking up with each new cohort more easily.

Feedback was collated at the end of each placement and was acted on in real time prior to the next cohort of students starting. There were 5 cohorts of students that were asked to provide feedback across 10 qualitative and quantitative questions within Microsoft Forms.

**Results:** Initial results showed 64% of students were dissatisfied with the induction process in the first rotation. 45% of people were unhappy with the bedside teaching. Organisation of the sub-specialities was not consistent across the trust. Both positives and negatives were highlighted and tracked. Trends of responses were monitored compared against both time and site (as students were placed across three sites) and acted upon to create an atmosphere of constant improvement and was correlated against informal conversations. After changes were made students were 50% more satisfied with the placement and standard of bedside teaching.

**Conclusion:** We acted on general themes from the feedback. We concluded that introducing an induction programme to explain placements and reiterate safety would improve the programme. There was increased time on the wards, direct patient contact and sub-speciality experiences. Feedback following changes showed improvement. Improvements in student-focused teaching were seen with better knowledge gained from small group teaching and 3 structured sessions included throughout. Core trainees will continue to reassess on a 5 weekly basis and make changes to the programme accordingly.

